# Emotional design of new energy vehicle center consoles for elderly users based on entropy weighted TOPSIS and BPNN

**DOI:** 10.1038/s41598-025-31226-4

**Published:** 2025-12-08

**Authors:** Hui Gui, Jibin Zhang, Wen Liu, Xiang Li

**Affiliations:** 1 School of Art and Design, Wuhan University of Technology, Wuhan, 430070 People’s Republic of China; 2 School of Information Management, Wuhan University, Wuhan, 430072 People’s Republic of China; 3 School of Art and Design, Wuhan Institute of Technology, Wuhan, 430205 People’s Republic of China

**Keywords:** New energy vehicles, Center console design, Elderly-friendly design, Kansei engineering, Entropy weight-TOPSIS, Backpropagation neural networks, Mechanical engineering, Engineering, Mathematics and computing

## Abstract

With the increasingly serious issue of global population ageing, meeting the emotional needs of elderly users when interacting with the center consoles of new energy vehicles (NEVs) has become significant. At present, the design of NEV center consoles presents difficulties for elderly users in terms of both operation and usability. To ensure driving safety and comfort, console design must take into account the operational convenience and psychological experience of elderly users. Therefore, to design a center console that meets the emotional needs of elderly users, this study proposes a comprehensive methodological framework based on Kansei Engineering (KE), the entropy-weighted technique for order of preference by similarity to an ideal solution (TOPSIS), and Backpropagation Neural Networks (BPNN), focusing on the elderly-oriented design of NEV center consoles. First, within the framework of KE, emotional evaluation indicators were collected and analysed based on the usage habits and Kansei requirements of elderly users. Second, entropy-weighted TOPSIS was applied to rank the morphological features of center consoles and to extract the key design features. Finally, a BPNN-based mapping model was constructed to link elderly users’ Kansei intentions with the critical design features of the center console. The experimental results show that, among the morphological configurations, the combination of center control screen Type II, co-driver entertainment screen Type II, control panel Type II, and center console outline Type II best satisfies the emotional needs of elderly users. By integrating KE, entropy-weighted TOPSIS, and BPNN, this study provides a more effective means of understanding and predicting user emotions, thereby supporting the design of NEV center consoles that better meet the emotional requirements of elderly users.

**Supplementary Information:**

The online version contains supplementary material available at 10.1038/s41598-025-31226-4.

## Introduction

Population aging will become one of the most significant social transformations of the 21 st century^1^. By 2050, the number of older adults and the youth population will be equal, with each group comprising 21% of the global population—2 billion people aged 60 and above, and 2 billion under the age of 15^2^. Providing safe, friendly, affordable, and convenient transportation for older adults is considered essential for enhancing their quality of life^3^. Currently, most researchers focus on the aesthetic design and performance enhancement of new energy vehicles (NEVs). For instance, LAI et al.^4^have proposed using Kansei engineering (KE) as a foundation, combining it with the Apriori + SEM method, to study the exterior design of NEVs. Kang^5^has established a mapping relationship between customer perceptions and HEV design features using rough set theory and support vector regression, achieving an optimal design integration. Yu et al.^6^. developed a human-machine hybrid intelligence methodology—integrating human cognitive mental models, a shared knowledge base, and Generative Adversarial Networks (GANs)—to create numerous car front designs that align with design intents by establishing consistency in human-machine cognitive structures, thus reducing communication barriers during the human-machine hybrid creative design process. Ostrosi et al.^7^. have utilized the full sub-concept of style, proposing a computational method to address the multi-scale style recognition problem in automobiles. Chen et al.^8^. have introduced a human-machine cooperative vehicular platooning control scheme for connected autonomous vehicles (CAV) to solve the external environment challenges of autonomous driving, thereby enhancing following performance. Some studies have also focused on the interface design of new energy vehicles. For example, Chen^9^ analyzed automotive human-machine interface (HMI) within the framework of KE and established a mapping relationship between the emotional needs of elderly users and the key design features of HMI, thereby developing HMI designs that align with the emotional preferences of elderly users.

As autonomous driving technology advances, NEVs not only provide a safer and more convenient driving experience but also enable elderly users to drive for extended periods^10^. In this context, the elderly demographic is increasingly focusing on the emotional experience within the cabin of NEVs^5^. In fact, for drivers, the most direct and frequently contacted part of a vehicle is not its exterior but its interior design^11^, with the center console being a crucial component of this. Serving as the central hub for driver-vehicle interaction, the design of the center console not only affects the ease and safety of operations but also influences the user’s emotional experience and satisfaction. Current design trends in NEVs often overly emphasize the intelligence and modernity of operations, thus neglecting the actual needs and emotional experiences of elderly users regarding the center console.

As the elderly population grows, they are becoming a significant consumer segment. Particularly in the era of emotional consumption, elderly customers are focusing not only on the functionality of products but also on enjoyable experiences and personalized expression. Currently, the design of NEVs on the market is mostly aimed at younger consumers and often overly emphasizes functionality. The design of center consoles tends to overlook the emotional needs of the elderly, such as visual readability and ease of use, which poses challenges to the safety and comfort of elderly users. Therefore, the center console design of NEVs should address the emotional needs and sensory experiences of elderly users, providing more considerate and effective elderly-oriented design solutions. To this end, this study employs KE to research elderly-oriented design of NEV center consoles.

KE, introduced by the Japanese scholar Mitsuo Nagamachi in 1970, is a technology that transforms user emotional perceptions into product design features. Its core lies in quantifying users’ emotional needs and sensory experiences^12^. KE was first applied in the automotive industry and achieved great success. For example, Nissan used KE for interior^13^ and taillight designs^14^, and the Mazda project team employed category classification methods and driving simulators in KE research to develop the sports car Miata^15^. Today, KE methods are widely used across various fields, including product design^16,17^ transportation vehicles^18,19^ fashion design^20^, electronic devices^21^, and service design^22^.

KE design methods require extensive questionnaires and interviews to collect data on users’ emotional preferences. These data are then used to identify the key design features of the samples and to establish a mapping model between user emotions and these features. The emotional vocabulary primarily captures users’ emotional needs and sensory experiences. Researchers like Huang et al.^23^, Zhao et al.^24^, and Lai et al.^25^ have utilized Grey Relational Analysis (GRA) to explore the importance of multiple influencing factors, selecting the option with the highest grey-weighted relational result as the optimal solution. Li and Dai^26^ employed the Delphi method to filter the spatial components of mobile exhibition spaces in community art galleries, thereby identifying critical components needed for the spatial structure. In the process of identifying key product morphological features, GRA demands high-quality data and may face issues with the subjectivity of parameter selection and result stability; the Delphi method relies on expert opinions, which may be biased and difficult to quantify. In contrast, the entropy-weighted technique for order of preference by similarity to an ideal solution (TOPSIS) combines entropy weighting for attribute weighting with TOPSIS for ranking alternatives. This multi-attribute decision-making method reduces subjectivity and complexity, producing more objective and scientifically reliable results.

The essence of KE lies in establishing a mapping relationship between user emotions and the morphological features of a product. Traditional linear statistical methods, including correlation coefficient analysis, principal component analysis (PCA), factor analysis (FA), and multiple linear regression analysis (MLR), are primarily designed to elucidate linear relationships. However, they often fail to accurately represent the nonlinear and complex interactions between user emotions and product features, nor can they effectively adapt to variations in data structures. Therefore, with the advancement of artificial intelligence, an increasing number of machine learning techniques, such as Backpropagation Neural Networks (BPNN)^27^, interactive genetic algorithms (IGA)^28,29^, support vector regression (SVR)^30^, artificial neural networks (ANN)^31^, and long short-term memory networks (LSTM)^32^, have been explored for developing models that map user perceptions to design features. BPNN is a classic artificial neural network model with strong nonlinear adaptive information processing capabilities^33^. It simulates the human neural network learning process to construct mapping relationships in a nonlinear manner and has been widely used to assist in generating design decisions^34^.

Therefore, this paper proposes a design process that integrates KE, entropy weighted-TOPSIS, and BPNN, aimed at developing a NEV center console that meets the emotional needs of elderly users. Currently, research on the design of NEV center consoles primarily focuses on technical performance and aesthetics, with relatively less attention paid to elderly-oriented designs tailored to the needs of the aging population. While BPNN is widely used in pattern recognition and prediction, its application in elderly-oriented design is still in the early stages.

The design of NEV center consoles, tailored for elderly users, not only fulfills their emotional requirements, enhancing travel usability and comfort, but also offers theoretical guidance for both the NEV industry and its designers. Moreover, it expands the elderly consumer base and promotes sustainable societal development. The main contributions of this paper can be summarized as follows:

1. Currently, there is limited research on center console designs for elderly users in NEVs.
2. Previous KE research often did not fully integrate advanced data analysis techniques, and there has been little design research combining the entropy weighted-TOPSIS with BPNN methods.
3. The entropy weighted-TOPSIS method simplifies the center console design features, reducing the impact of subjective factors, making evaluation results more objective and accurate, and enhancing decision-making efficiency and quality.
4. BPNN replaces traditional linear analysis methods to establish a mapping relationship between user perceptions and NEV center console designs.

The remaining structure of this paper is as follows: Chap. 2 reviews the key concepts of the methodologies employed in the study; Chap. 3 presents the research framework for the design of NEV center consoles based on the emotional needs of elderly users; Chap. 4 analyzes and discusses the research findings; Chap. 5 provides a summary of the paper’s key contributions and limitations, while also presenting recommendations for future research directions.

## Related methods

### Application of KE in center console design

With the arrival of the emotional era, customers are no longer solely concerned with the usability and accessibility of products, but also with the emotional experience and aesthetic value they provide^35^. KE explores the relationships between users’ emotional appeals, emotional perceptions, and product design features, transforming users’ emotional experiences with products into quantifiable design elements^36^. Thus, in the new product development process, designers need to deeply understand customers’ psychological and emotional needs, and effectively integrate these subjective perceptions into product design. In current research, Lin et al.^37^ explored drivers’ emotional perceptions of the vehicle center console from the perspective of KE and identified the attraction factors and features that influence consumers’ preferences for vehicle center consoles. Lee et al.^38^ focusing on automotive interior design, employed KE methods to study the impact of multisensory and unisensory experiences on drivers’ perceptions, finding that metallic control panels and leather seats are respectively perceived as conveying a sense of technology and leisure. Ren et al.^39^ used KE methods and multiple linear regression analysis to establish a predictive model linking adjectives that describe dashboard shapes with perceived images. This approach converted drivers’ emotional perceptions into design elements and enhanced the alignment between dashboard design and emotional needs. Li et al.^40^ utilized techniques such as BP neural networks, GA-BP, SVM, and GA-SVM to build a model between the design elements of automotive interior colors and user evaluations, providing theoretical support for the subjective evaluation of interior color designs. Jindo et al.^13^ focused on the dashboard and steering wheel in automotive interiors, subjectively evaluating the relationship between emotional perception of these elements and their features.

Although KE has been widely applied in automotive interior design, research focusing specifically on the center console area remains relatively scarce. Most studies concentrate on the overall harmony, comfort, and functionality of the interior, neglecting the specific needs of the center console as the core area of interaction between the driver and the vehicle. Moreover, existing research seldom considers the diverse emotional needs of the elderly regarding center console design. Therefore, this study takes the center console of NEVs as a case study, with elderly users as the target group. The aim is to develop center console designs tailored for elderly users, promote elderly-oriented design, enhance societal attention to this demographic, and foster sustainable societal development.

### Entropy weight-TOPSIS theory

The entropy weight-TOPSIS method is an integrated evaluation technique that combines the entropy weight method and the TOPSIS method, two decision analysis tools. This approach allows for a more comprehensive and accurate assessment of issues, making the decision-making process more scientific and rational^41^.

The entropy weight method is an objective weighting method based on the concept of information entropy, used in multi-criteria decision analysis to determine the weights of various evaluation indicators. It is characterized by strong operability and objectivity, and it is capable of reducing biases from subjective evaluations^42^. The method assesses the dispersion of indicator values to reflect the amount of information an indicator provides, thereby determining its relative importance in the composite evaluation. In multi-criteria decision analysis, an indicator with a smaller information entropy indicates a greater range of data variation and thus provides more useful information, warranting a higher weight. Conversely, if an indicator’s information entropy is higher, indicating more concentrated data with less informational content, it should be assigned a lower weight^43^.

TOPSIS is a multi-attribute decision analysis method^44^, which was proposed by Hwang and Yoon in 1981,primarily used to select the optimal alternative from multiple options. In a multi-attribute decision-making environment, each alternative exhibits performance across various attributes. TOPSIS evaluates and selects the optimal solution based on the concept of distance. A solution is considered superior when it is closer to the positive ideal solution and farther from the negative ideal solution compared to other alternatives^45^..

The entropy weight-TOPSIS method differs from other multi-criteria decision-making (MCDM) approaches through its unique combination, which ensures the automatic determination of criteria weights throughout the process, thereby minimizing the impact of subjective human factors^46^. The calculation steps are as follows:

Create a mean evaluation matrix, where $\:{x}_{ij}$ represents the value of the $\:i$th scheme for the $\:j$th indicator; normalize the mean matrix to obtain a standardized matrix; and use the standardized matrix to calculate the weight $\:{P}_{ij}$ of each scheme for each indicator using the following formula:

1 $$\:{P}_{ij}=\frac{{x}_{ij}}{\sum\:_{i=1}^{n}\:{x}_{ij}}$$

Compute the information entropy of each indicator. The information entropy reflects the dispersion of the indicator. $\:{E}_{j}$ represents the information entropy of the $\:j$th indicator, when $\:{P}_{ij}=0$, $\:{P}_{ij}\mathrm{ln}\left({P}_{ij}\right)=0$, the calculation formula is as follows:

2 $$\:{E}_{j}=-\frac{1}{\mathrm{ln}\left(n\right)}\sum\:_{i=1}^{n}\:{P}_{ij}\mathrm{ln}\left({P}_{ij}\right)\:\:\:\:\:\:\:\:\:\:\:\left(j=\mathrm{1,2},\dots\:,m\right)$$

The difference coefficient $\:{d}_{j}$ and the weight $\:{w}_{j}$ of the evaluation index are calculated based on information entropy, where $\:{d}_{j}$ represents the information utility value of the $\:j$th index and $\:{w}_{j}$ is the weight of the $\:j$th index, and the calculation formula is:

3 $$\:{d}_{j}=\left|1-{E}_{j}\right|\:\:\:\:\:\:\:\:\left(j=\mathrm{1,2},\dots\:,m\right)$$

4 $$\:{w}_{j}=\frac{{d}_{j}}{\sum\:_{j=1}^{m}\:{d}_{j}}\:\:\:\:\:\:\:\left(j=\mathrm{1,2},\dots\:,m\right)$$

The TOPSIS method is used to rank the advantages and disadvantages of the solutions in this study by incorporating the weight coefficients of each indicator. The normalized decision matrix $\:{(z}_{ij})$ is calculated to measure the distance from the positive and negative ideal solutions, as shown in the following formula:

5 $$\:{z}_{ij}=\frac{{x}_{ij}}{\sqrt{\sum\:_{i=1}^{n}\:{x}_{ij}^{2}}}$$

Use Eqs. (6) and (7) to calculate the positive ideal solution $\:{Z}^{+}$ and the negative ideal solution $\:{Z}^{-}$.

6 $$\:\left.{Z}^{+}=\left\{\underset{1\leqslant{i}\leqslant{m}}{max}\:{z}_{ij}\right\}\mid\:J=\mathrm{1,2},\cdots\:,n\right\}=\left\{{Z}_{1}^{+},{Z}_{2}^{+},\cdots\:{Z}_{n}^{+}\right\}$$

7 $$\:\left.{Z}^{-}=\left\{\underset{1\leqslant{i}\leqslant{m}}{min}\:{z}_{ij}\right\}\mid\:J=\mathrm{1,2},\cdots\:,n\right\}=\left\{{Z}_{1}^{-},{Z}_{2}^{-},\cdots\:{Z}_{n}^{-}\right\}$$

Calculate the distance between the evaluation index and the positive or negative ideal solution. $\:{w}_{j}$ is the weight of the $\:j$th index. Equation (8) is used to determine the distance between each alternative and both the positive ideal solution and the negative ideal solution.

8 $$\:\left\{\begin{array}{c}{D}_{i}^{+}=\sqrt{\sum\:_{j=1}^{m}\:{w}_{j}{\left({Z}_{j}^{+}-{z}_{ij}\right)}^{2}};\\\:{D}_{i}^{-}=\sqrt{\sum\:_{j=1}^{m}\:{w}_{j}{\left({Z}_{j}^{-}-{z}_{ij}\right)}^{2}};\end{array}\:\:\left(i=\mathrm{1,2},\dots\:,m;j=\mathrm{1,2},\dots\:,n\right)\right.$$

Calculate the relative proximity factor $\:{C}_{i}$, and finally sort the schemes according to the value, where a larger $\:{C}_{i}$ value indicates a better scheme.

9 $$\:{C}_{i}=\frac{{D}_{i}^{-}}{{D}_{i}^{+}+{D}_{i}^{-}}\left(i=\mathrm{1,2},\cdots\:,m,0\leqslant{C}_{i}\leqslant1\right)$$

This study incorporates the entropy weight method into the TOPSIS decision-making framework, effectively combining the weighting capabilities of entropy values with the ranking efficiency of the TOPSIS method. The combination of the entropy weight method and TOPSIS provides a more comprehensive and accurate evaluation. The entropy weight method objectively determines the weights of indicators through the calculation of information entropy, while TOPSIS evaluates the merits of alternatives by comparing their distances to an ideal solution. Salehi et al.^47^. applied the entropy-weighted TOPSIS method to assess crisis management systems in the petrochemical industry. They ranked the systems of surveyed factories, and the results provided insights that can help managers and decision-makers improve crisis management practices in the petrochemical sector. Li et al.^48^ used the TOPSIS method to evaluate land use performance in the Shunyi District from 1996 to 2010, determined the weights of the indicators using the entropy weight method, and analyzed the key factors affecting land use performance using grey relational analysis, providing methods and ideas for comprehensive evaluation and optimal allocation of regional land resources. Satı et al.^49^studied global indices such as the Global Innovation Index (GII), the Global Competitiveness Index (GCI), and the Network Readiness Index (NRI), analyzing these data with the entropy-TOPSIS method, comparing the EU and candidate countries, and finding that Austria, Denmark, and Germany ranked among the top three. Wang et al^50^. employed the multi-criteria decision-making method, entropy weight-TOPSIS, to evaluate 22 co-generation technologies in the steel industry, setting six categories of criteria to comprehensively assess the overall performance of each technology.

### Backpropagation neural network

**Fig. 1:**
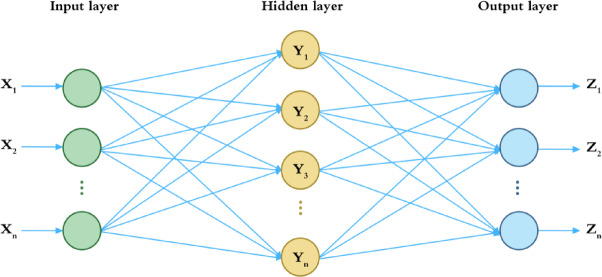
BPNN model structure diagram.

BPNN is a multilayer feedforward neural network with powerful nonlinear mapping capabilities, suitable for establishing predictive relationships between users’ emotional needs and product design features^51^. The structure of the BPNN model is shown in Fig. 1. Owing to its advantages in pattern recognition and regression analysis, BPNN has been widely applied in product optimization design^52^. Wang et al.^53^ utilized BPNN to identify relationships between design variables and user emotional needs, thus constructing a predictive model. Misaka and Aoyama^54^ combined KE and BPNN to develop a design system capable of producing cup surface crack patterns that met user expectations. Li et al.^55^employed emotional ergonomics and BPNN to predict the form features of footwear products. Lin et al^56^. used emotional ergonomics and a BPNN based on genetic algorithms to establish a predictive model linking the design elements of electric shaver sounds with users’ emotional evaluations. This approach further explored the emotional connotations conveyed by the sounds of electric shavers. Guo et al^57^. focused on mobile phones, designing an optimization algorithm based on genetic algorithms and BPNN, and established a mobile phone optimization design model that considered both design and user emotional requirements. Deng et al.^58^ combined KE with BPNN and, by leveraging the complex nonlinear mapping capability of BPNN, established a predictive model for the emotional imagery of colors in human–machine interaction interfaces.

Consequently, this paper employs BPNN to construct a mapping between the emotional intentions of elderly users and the design features of the center console. The specific steps are as follows:

BPNN is structured with an input layer, an output layer, and several hidden layers. The input layer contains $\:N$ nodes, each corresponding to a design feature, while the output layer comprises $\:K$ nodes, each representing an emotional term. $\:M$ denotes the number of nodes in the hidden layers. The formula can be described as follows:

10 $$\:M=\frac{N+K}{2}$$

BPNN employs the Trainlm (Levenberg-Marquardt) gradient descent algorithm, with the output parameters constrained between [0,1]. To facilitate this, emotional evaluations require normalization. The Min-Max Normalization method is used for both input and output normalization, where $\:{x}_{max}$ and $\:{x}_{min}$ are the maximum and minimum values, respectively. This normalization improves the convergence and efficiency of network training. The normalized data for both input and output variables, $\:{x}_{\alpha\:}$, is computed using the following formula:

11 $$\:{X}_{\alpha\:}=\frac{{x}_{\alpha\:}-{x}_{min}}{{x}_{max}-{x}_{min}}$$

The output parameters can be fed into the BPNN model for training. The activation function for the hidden layers is typically the logistic sigmoid transfer function, which is represented as follows:

12 $$\:f\left(x\right)=\frac{1}{1+{e}^{-x}}\:\left(0<f\left(x\right)<1\right)$$

The output layer uses the linear activation function purelin. The performance of the model is evaluated by calculating the standard deviation by examining the root mean square error (RMSE) between the predicted value and the true value. The commonly used expression for RMSE is as follows:

13 $$\:\mathrm{R}\mathrm{M}\mathrm{S}\mathrm{E}=\sqrt{\frac{\sum\:_{i=1}^{n}\:{\left({x}_{i}-{x}_{0}\right)}^{2}}{n}}$$

### Ethical statement

We confirm that all methods were carried out in accordance with relevant guidelines and regulations. All experimental protocols were approved by the Academic Committee of Wuhan Institute of Technology, and informed consent was obtained from all participants and/or their legal guardians.

## Research process and results

### Proposed research framework

**Fig. 2:**
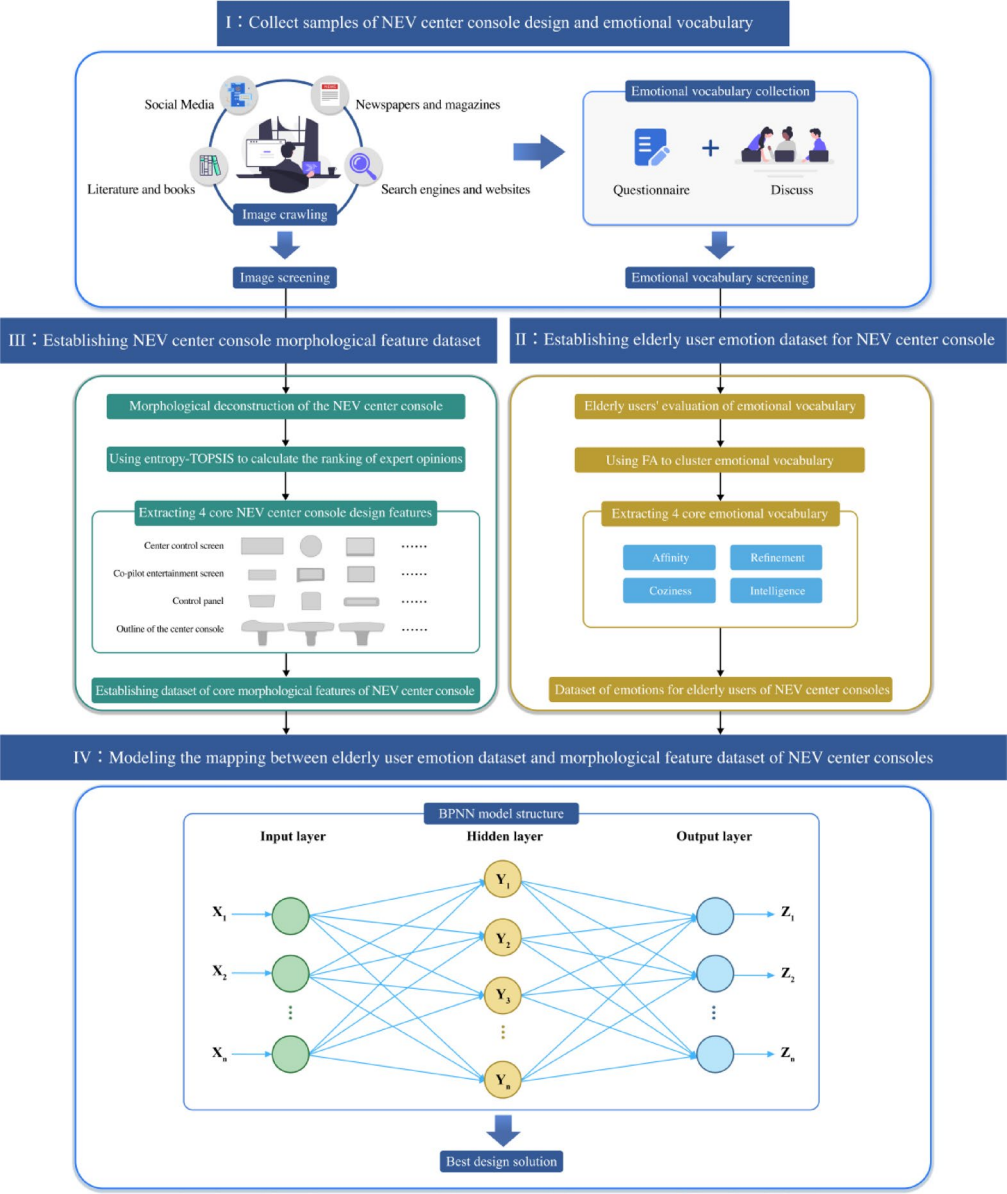
Research framework diagram.

This paper develops an age-friendly design for the center console of NEVs within the framework of KE, integrating entropy weighted-TOPSIS and BPNN. First, Python programming was employed to collect samples of NEV center consoles from automotive websites, and a representative sample set was established after screening. At the same time, emotional vocabulary was compiled through a review of literature and online sources, supplemented by an emotional intention evaluation questionnaire. Following expert screening, 10 Kansei words were identified. To reduce information load and uncover potential consistency among these words, factor analysis was applied to perform dimensionality reduction and simplification. As a result, four core Kansei words representing elderly users were determined, and an emotional dataset was established. Subsequently, morphological decomposition was conducted on 60 collected samples, producing seven design morphological features. The entropy-weighted TOPSIS method was then employed to filter and rank the morphological dataset, eliminating categories with minor influence and identifying four key morphological features. On this basis, a key morphological dataset was constructed. Finally, a BPNN was employed to develop a predictive model that maps the emotional intentions of elderly users to the critical design features of the center console. This model facilitates the determination of the optimal age-friendly design combination for the center console of NEVs. The specific research framework is shown in Fig. 2.

### Collection of morphological features and emotional vocabulary samples of NEV center consoles

**Fig. 3:**
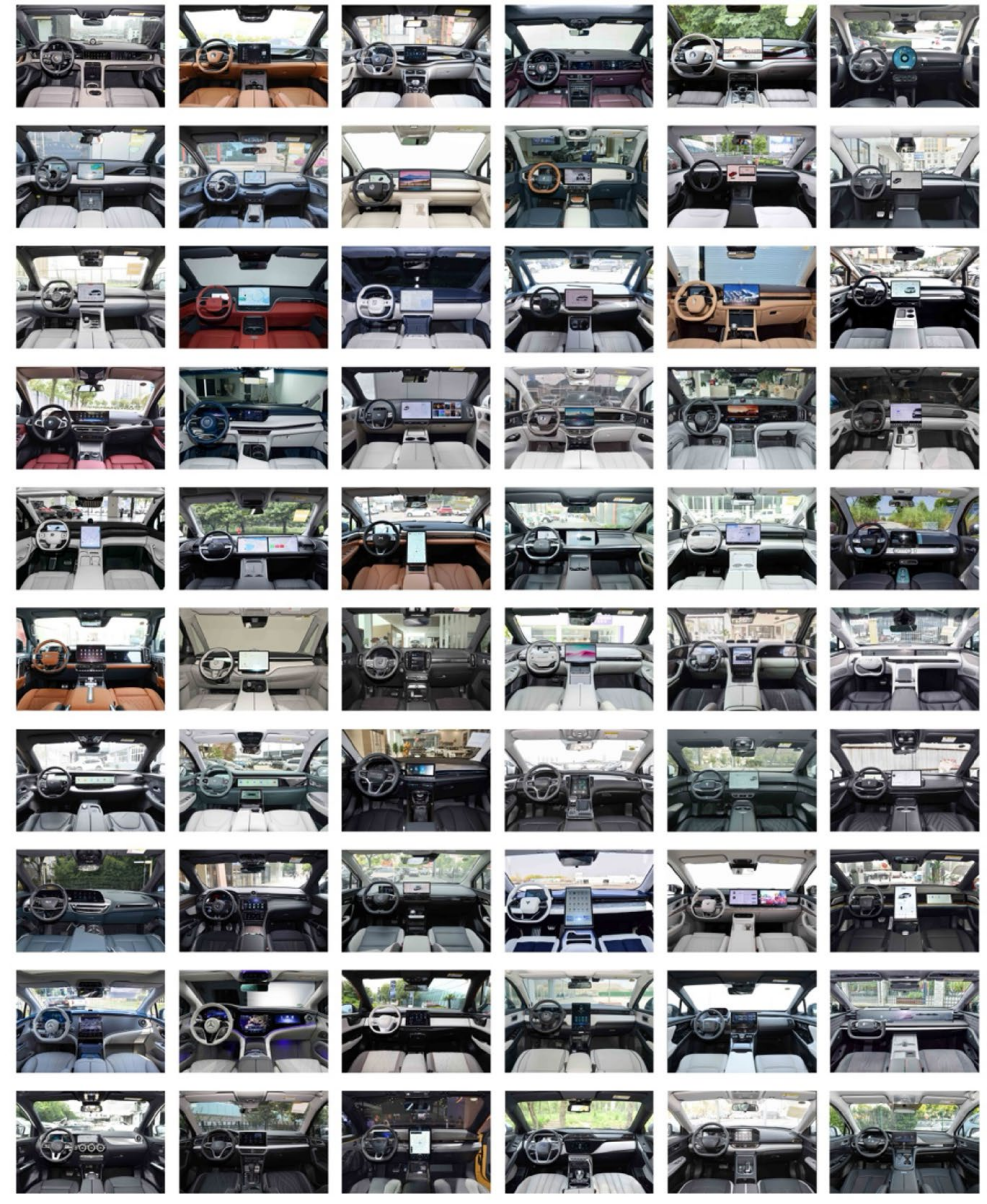
Collection of 60 NEV center console samples.

In this study, the front view of the center console design was used as the sample perspective. Using Python programming and keywords such as “car center console” and “car interior,” 100 samples were collected from automotive websites. After discussions in focus groups, images that were blurry, obstructed, distorted in angle, or overly familiar were discarded. Ultimately, 60 representative center console images were selected to establish a sample dataset for NEV center consoles, as shown in Fig. 3.

Through a comprehensive review of literature, magazines, and online resources, and the simultaneous distribution of an emotional intention evaluation questionnaire for the NEV centre console samples, a total of 80 adjectives were collected to describe the emotional feelings towards the centre console. The emotional vocabulary collected in this study was not related to whether the emotions were positive or negative, but focused on users’ emotional needs and sensory experiences. During the experiment, the subjects only evaluated the shapes through static pictures and did not come into contact with any tasks or prompts related to voice or touch control. Therefore, these factors did not affect the present emotional evaluation results. Subsequently, five experts with experience in automotive interior design were invited to screen these 80 emotional words. We eliminated meaningless words and combined those with similar meanings. Finally, we selected 10 representative emotional words, as shown in Table 1.

**Table 1:** 10 emotional words of NEV center Consoles.

10 emotional words of NEV Center Consoles				
Comfortable	Simple	Practical	Friendly	Cozy
Technological	Elegant	Bright	Innovative	Advanced

### Establishing an emotional design dataset for NEV center consoles

#### Creation of elderly user emotional dataset for NEV center consoles

**Fig. 4:**
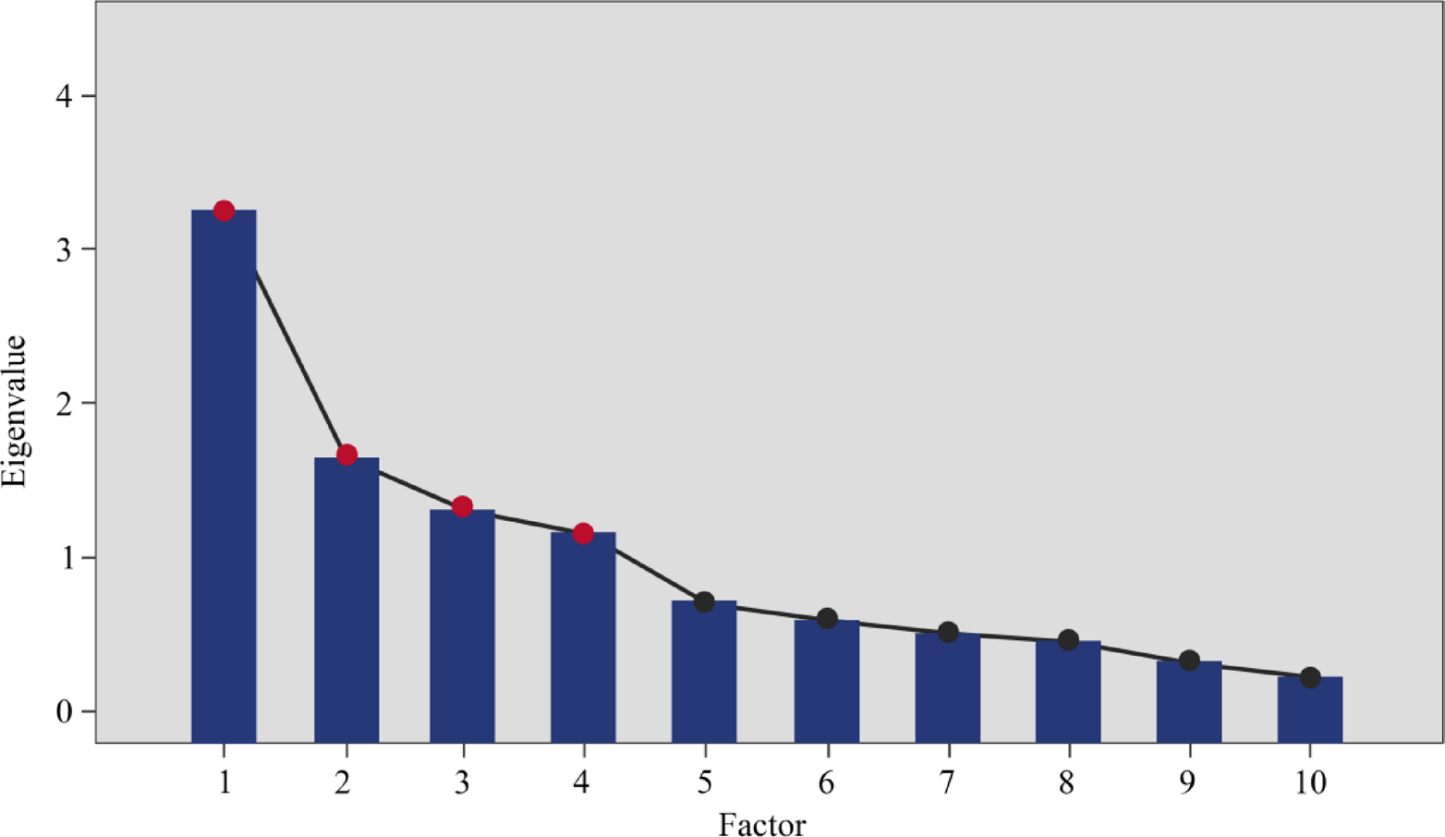
Scree plots.

The 10 representative emotional terms selected above were used in a questionnaire survey employing a seven-point Likert scale, distributed to 157 participants, to evaluate 60 samples of new energy vehicle center consoles across 10 emotional intention categories. A total of 153 valid questionnaires were returned, yielding a response rate of 97%. To ensure the objectivity of the survey results, the average emotional vocabulary score for each sample was calculated statistically, with the average scores shown in Supplementary Table S1. The average emotional vocabulary values for each sample were then imported into IBM SPSS Statistics (Version 29.0; https://www.ibm.com/products/spss-statistics). To mitigate information overload and reveal the latent structure among Kansei words, this study employed Exploratory Factor Analysis (EFA) to perform dimensionality reduction on the emotional evaluation matrix data. It is important to emphasize that the primary objective of using EFA in this research was to serve as a data preprocessing step for the subsequent BPNN prediction model. Specifically, Varimax rotation was utilized to generate mutually uncorrelated (orthogonal) factors to enhance the stability and predictive performance of the neural network training. This approach effectively eliminates multicollinearity among target vectors, thereby providing the model with clear, non-redundant target signals. Arkadiusz et al.’s^59^ research indicates that during factor analysis, it is necessary to first perform Kaiser-Meyer-Olkin (KMO) and Bartlett’s sphericity tests on the scale data to verify whether the structural reliability and validity of the scale meet the basic research standards for factor analysis. The experimental results are shown in Table 2. The KMO value was 0.678 (> 0.6), Bartlett’s sphericity test was 177.433, the degrees of freedom were 45, and the significance value was < 0.001 (When the KMO value is greater than 0.5 and the Bartlett’s sphericity test p-value is less than 0.05, the data are deemed suitable for factor analysis). These data are statistically significant. In summary, the KMO and Bartlett’s sphericity test confirm that the data is suitable for factor analysis. In the scree plot (Fig. 4) and total variance (Table 3), there are four factors with eigenvalues greater than 1, indicating that the dataset can be reduced to four factors. The cumulative contribution rate of the four reduced factors reaches 73.162%. The rotated factor matrix is shown in Table 4. To reduce visual interference, factors with absolute values less than 0.5 are represented by blank cells.

**Table 2:** KMO and Bartlett sphericity test.

KMO		0.678
Bartlett sphericity test	Approximate chi-square value	177.433
	Freedom	45
	Significance	< 0.001

**Table 3:** Total variance explanation.

Initial eigenvalue				Extract the sum of squares			Rotational load sum of squares		
Ingredient	Total	Percentage of variance	Cumulative%	Total	Percentage of variance	Cumulative%	Total	Percentage of variance	Cumulative%
1	3.249	32.488	32.488	3.249	32.488	32.488	2.602	26.017	26.017
2	1.633	16.335	48.823	1.633	16.335	48.823	1.792	17.924	43.941
3	1.294	12.938	61.761	1.294	12.938	61.761	1.566	15.656	59.598
4	1.140	11.400	73.162	1.140	11.400	73.162	1.356	13.564	73.162
5	0.686	6.855	80.017						
6	0.575	5.746	85.763						
7	0.490	4.898	90.661						
8	0.434	4.339	95.000						
9	0.300	2.999	97.999						
10	0.200	2.001	100.000						

**Table 4:** Rotated component matrix.

	1	2	3	4
Comfortable	0.753			
Simple	0.764			
Practical	0.853			
Friendly	0.779			
Cozy			0.867	
Technological				0.829
Elegant		0.862		
Bright			0.851	
Innovative				0.792
Advanced		0.851		

The component score coefficient matrix obtained using principal component analysis is shown in Table 5. Based on the above experimental results, four main factor components can be extracted. The naming of the factors and the selection of indicators were based on a comprehensive consideration of the emotional needs of the elderly. The first factor consists of four indicators: comfort, simplicity, practicality, and friendliness. These four indicators are designed to make users feel relaxed, comfortable, and happy when using the product, and are therefore called the “affinity” factor. The second factor is composed of the indicators of elegance and advanced, which together convey a refined and high-quality emotional experience, hence named the “Refinement” factor. The third factor is composed of the indicators of coziness and brightness, hence named the “Coziness” factor. The fourth factor is composed of “technological” and “innovative,” with both indicators reflecting the product’s cutting-edge technology and design, hence the name “Intelligence” factor. These four factors correspond to the Affinity, Refinement, Coziness, and Intelligence dimensions reflected in the design of the NEV center console, thereby establishing an emotional structure for the design of the central control panel in new energy vehicles based on the emotional needs of elderly users.

**Table 5:** Component score coefficient matrix.

	1	2	3	4
Comfortable	0.372	−0.202	0.107	−0.072
Simple	0.311	−0.029	0.040	0.003
Practical	0.349	0.009	0.089	0.011
Friendly	0.290	0.066	0.013	−0.027
Cozy	0.065	0.096	0.593	0.062
Technological	0.036	−0.172	0.073	0.631
Elegant	−0.071	0.513	0.043	−0.069
Bright	0.114	−0.094	0.586	−0.029
Innovative	−0.101	0.120	−0.040	0.586
Advanced	−0.046	0.487	−0.039	0.013

Using the four most representative core emotional words extracted, a 7-point Likert scale was constructed and combined with the 60 samples of NEV center consoles. The questionnaire was redistributed to 104 individuals for emotional vocabulary intention scoring, thereby constructing an emotional dataset for elderly users of NEV center consoles (Supplementary Table S2).

#### Construction of key design features dataset for NEV center consoles

To obtain the key design features dataset for NEV center consoles, the first step involved categorizing and deconstructing the morphology of 60 NEV center console samples to extract key design morphologies that significantly impact elderly users. Subsequently, to simplify the input for the BPNN model and focus on the most critical design elements, the entropy-weighted TOPSIS method was employed for feature selection. This method aims to objectively rank the influence of the seven morphological categories based on expert evaluations, thereby identifying key design features rather than extracting latent factor structures. Ultimately, by selectively retaining the top-ranked key morphological categories, the key design features dataset for NEV center consoles was constructed.

##### Establish a morphological decomposition table for the center console of NEV

**Fig. 5:**
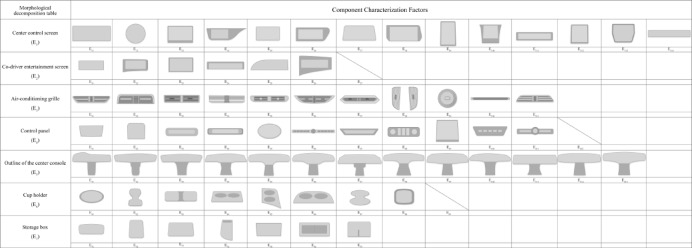
Morphological decomposition table of the NEV center console.

Through discussions with focus groups, the morphology of the center consoles is decomposed into seven categories: the center control screen (E1), co-driver entertainment screen (E2), air-conditioning grille (E3), control panel (E4), center console outline (E5), cup holder (E6), and storage box (E7). The structural table of morphological features for NEV center consoles is shown in Fig. 5.

##### Employing entropy weight-TOPSIS to investigate expert opinions for key morphological feature extraction

A panel of 20 specialists was convened, comprising eight designers skilled in NEV interior aesthetics and twelve graduate supervisors in industrial design. The experts evaluated the influence of seven morphological categories on elderly users’ emotions using a seven-point Likert scale, resulting in the construction of an average matrix, presented in Table 6.

**Table 6:** Impact of center console components on elderly users’ emotions in NEV.

Component category	Affinity	Refinement	Coziness	Intelligence
Center control screen(E1)	4.9	4.85	5	6.1
Co-driver entertainment screen(E2)	5.05	4.7	4.75	5.25
Air-conditioning grille(E3)	4.5	4.6	4.4	5.1
Control panel(E4)	4.8	5	5	5.8
Center console outline(E5)	5.25	5.5	5.35	5.6
Cup holder(E6)	4.25	4.4	4.45	4
Storage box(E7)	4.3	4.3	4.45	4

Employing Eq. (1), the mean matrix underwent normalization to derive a standardized matrix. Thereafter, through the application of Eqs. (2) and (3), both the entropy values and weighting parameters for the emotional vocabulary associated with the center console were computed. This process facilitated the determination of information entropy values, utility values of information, and weighting values, as delineated in Table 7.

**Table 7:** Component weight calculation for the center console of a NEV.

Category	Information entropy value	Information utility value	Weight value
Affinity	0.813	0.187	23.157
Refinement	0.814	0.186	23.083
Coziness	0.754	0.246	30.58
Intelligence	0.813	0.187	23.18

The weighting parameters corresponding to the emotional vocabulary of the center console were assimilated within the framework of the TOPSIS methodology, encompassing seven evaluative subjects, each characterized by four attributes of emotional assessment. The attributes pertaining to each morphological element underwent vector normalization as per formula (4), culminating in the construction of a decision matrix. Subsequently, employing formulas (5 through 9), the optimal and least favorable solutions for each morphological variant of the center console were determined, along with the computation of the distances of each indicator from these ideal and nadir solutions. As shown in Table 8, the comprehensive scoring order of the center console components is as follows: center console outline (E5) > center control screen (E1) > control panel (E4) > Co-driver entertainment screen (E2) > air-conditioning grille (E3) > cup holder (E6) > Storage box (E7).

Based on the results of the entropy weighted-TOPSIS calculations, morphological feature categories with a comprehensive score index below 0.5 were eliminated. Four key morphological features were retained: the center console outline (E5), center control screen (E1), control panel (E4), and co- driver entertainment screen (E2). These features were identified as the most critical in influencing user emotions and preferences.

**Table 8:** Comprehensive scoring of NEV center console components.

Component category	Positive ideal solution distance($\:{\varvec{D}}_{\varvec{i}}^{+}$)	Negative ideal solution distance($\:{\varvec{D}}_{\varvec{i}}^{-}$)	Composite score index	Sequence
Center control screen(E1)	0.3709459	0.70718344	0.65593562	2
Co-driver entertainment screen(E2)	0.52135516	0.54542575	0.51128188	4
Air-conditioning grille(E3)	0.78642585	0.30413715	0.27888086	5
Control panel(E4)	0.36497411	0.66398662	0.64529831	3
Center console outline(E5)	0.11463153	0.95013742	0.8923414	1
Cup holder(E6)	0.96529219	0.04949814	0.04877672	6
Storage box(E7)	0.97266584	0.03776288	0.03737313	7

#### BPNN establishes a mapping model between Kansi semantics and key design features of the NEV center console

Based on the four core emotional words identified and the four key morphological features selected, a matrix was constructed to represent the relationship between the design features of the NEV center console and the Kansei evaluation values. This matrix is displayed in Supplementary Table S3.

In MATLAB 2023b, the BPNN was employed to train the affective semantic evaluation matrix presented in Supplementary Table S3. The training parameters for the BPNN are outlined in Table 9. The effectiveness of the network training is assessed by examining the errors in both the testing and training sets, allowing for an evaluation of the model’s performance and accuracy in predicting emotional responses based on design features of the center console.

**Table 9:** Parameters for BPNN training.

Parameter name	Parameter
Training function	Trainlm
Hidden layer activation function	logsig
Output layer activation function	purelin
Training set samples	No. 1~55
Test set samples	No. 56~60
Number of iterations or learning epochs	500

Using the emotional word “intelligence” as an example, the input layer of the BPNN consists of the four key design features that have been identified. The output layer contained one node, which corresponded to the emotional word “intelligent.” According to the BPNN formula (10), the number of nodes in the hidden layer was calculated to be 2.5. Next, the first 55 samples are used as the training set for input into the network. The network was set to undergo 500 learning iterations with a learning rate of 0.01. This configuration aims to optimize the network’s ability to model the relationship between design features and the emotional response of “intelligence,” ensuring that the network effectively learns and generalizes from the training data.

**Fig. 6:**
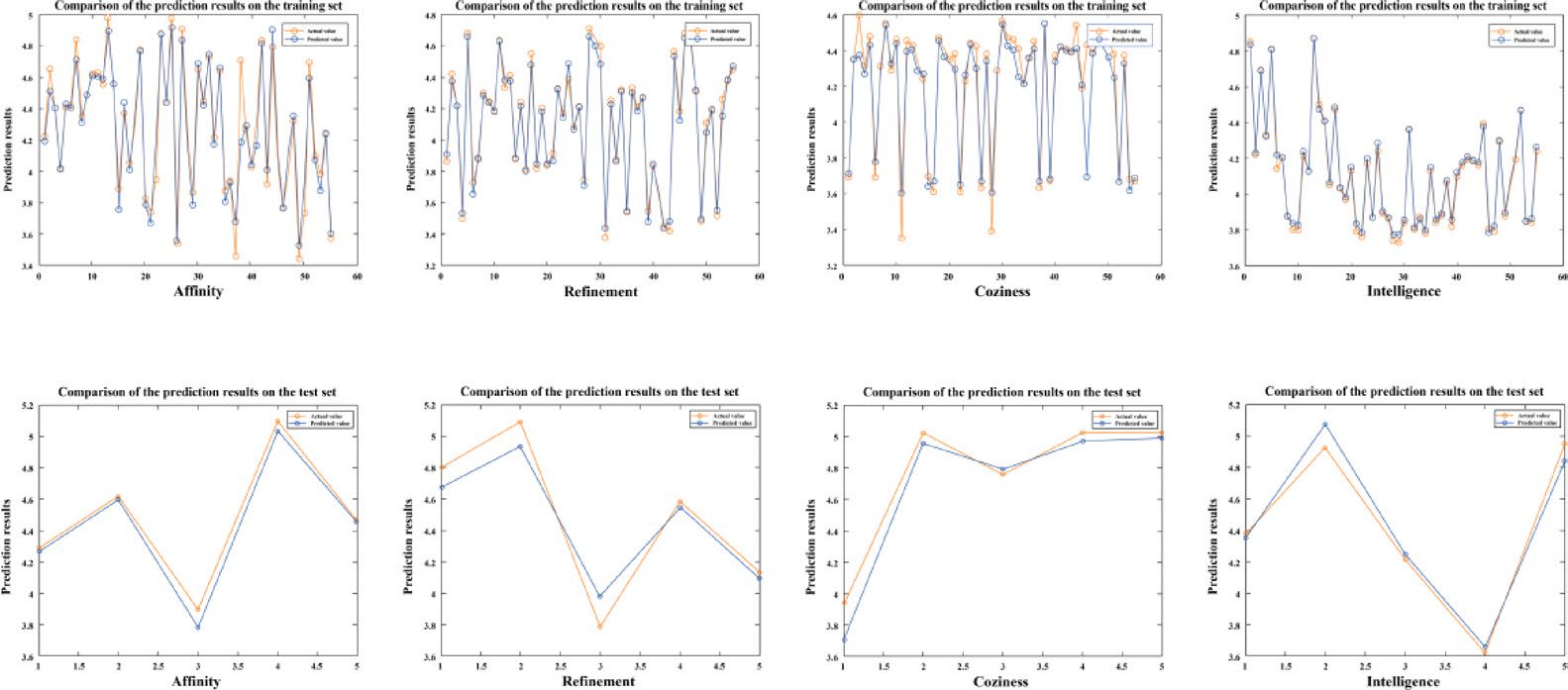
Fitting of the training and test sets.

**Fig. 7:**
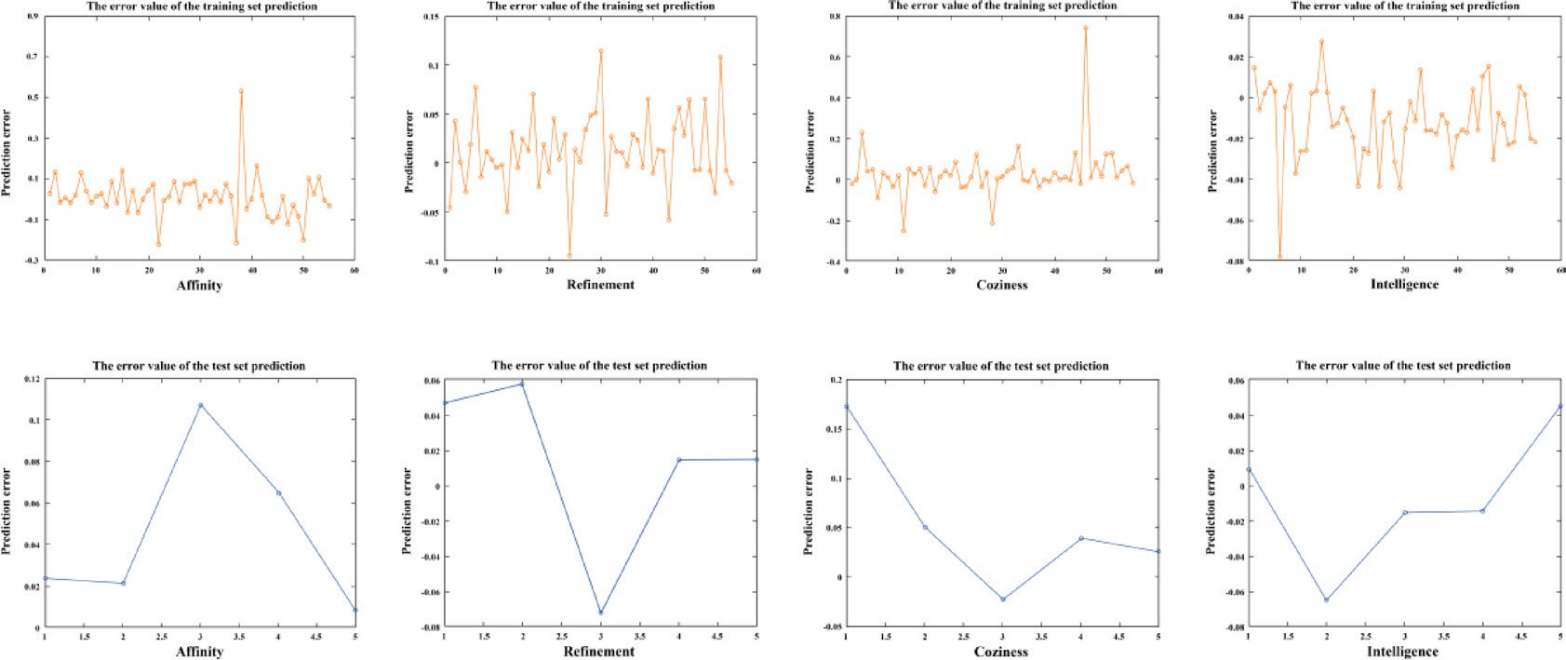
Error between training set and test set prediction values.

The hidden and output layers of the model employ the logsig and purelin functions, respectively, and are trained using the Trainlm gradient descent function. After multiple training iterations, the model converged to yield satisfactory predictive results for the training set. Taking the emotional word “intelligence” as an example, the model converged with favorable parameters (RMSE = 0.021937, R^2^ = 0.99405, MSE = 0.00048124), demonstrating strong predictive capabilities, high accuracy, and minimal deviation between predicted and actual values. Subsequently, samples 56 to 60 from Supplementary Table S3 were used to validate the reliability of the trained model. The comparison between actual perceived evaluation values from the test set and the network’s predicted values resulted in parameters (RMSE = 0.037237, R^2^ = 0.97043). These results confirmed that the model effectively and accurately assessed emotional responses, as detailed in Table 10. Based on this, the parameter results for the three emotional words “Affinity,” “Refinement,” and “Coziness” are obtained (Table 10); a comparison of the predictive results for the training and test sets is shown (Fig. 6); and the errors between the predicted values for the training and test sets are illustrated (Fig. 7).

**Table 10:** The parameter results.

Kansei word	The predicted results on the training set.		The result of the actual value and the predicted value.	
	*R* ^2^	RMSE	*R* ^2^	RMSE
Affinity	0.92128	0.10809	0.9803	0.057887
Refinement	0.98724	0.041072	0.87646	0.047595
Coziness	0.84235	0.12511	0.94933	0.084724
Intelligence	0.99405	0.021937	0.97043	0.037237

**Fig. 8:**
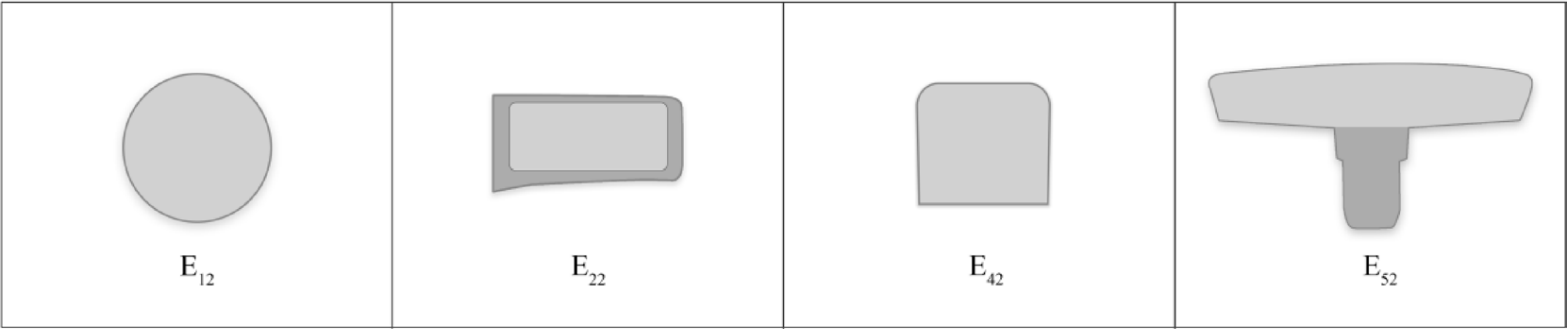
The best combination of design features.

By calculating the maximum average value of elderly users’ emotional word evaluations, the center console design feature combination with the highest degree of age-friendliness can be determined. After ranking using the entropy weighted-TOPSIS method, four key design features are identified. Each design feature combination consists of 14, 7, 12, and 13 elements respectively, resulting in a total of 14 × 7 × 12 × 13 = 15,288 combination design options. All combinations are computationally encoded to serve as input parameters for the BPNN model, which calculates the perceived values for each design option. The average value of the evaluations for the four emotional words in each sample is calculated, and the highest average value is selected as the basis for determining the highest degree of elderly-oriented design. The greatest average in the emotional evaluation is 4.472775, corresponding to the design feature combination of 2, 2, 2, 2. This combination is identified as the optimal design for an elderly-oriented center console. The combination of 2, 2, 2, 2 in the morphological feature deconstruction table corresponds to the 2nd center control screen, the 2nd co-driver entertainment screen, the 2nd control panel, and the 2nd center console outline, as illustrated in Fig. 8.

## Results analysis and discussion

In recent years, as the global population continues to age, industries are actively exploring how to provide older users with more convenient, comfortable, and safe products and services. Age-friendly design has increasingly become a central issue in the field of design. However, existing research largely relies on qualitative descriptions and lacks systematic quantitative analysis methods, resulting in relatively weak foundations for design evaluation. The key to age-friendly design lies not only in meeting the basic usage needs of the elderly but also in addressing their emotional needs. It must also consider their physiological characteristics and cognitive abilities, thereby supporting sustainable social development. Currently, there is relatively little systematic research on the combination of age-friendly design features in the field of new energy vehicle center consoles. Therefore, this study introduces entropy-weighted TOPSIS and BPNN within the framework of affective KE to quantitatively analyze and identify the optimal age-friendly design scheme for center consoles, thereby more effectively meeting the emotional needs of elderly users.

This paper took the central control panel of new energy vehicles as the research object. Through FA, the collected emotional intention vocabulary was reduced in dimension, and four representative emotional vocabulary terms—“affinity, refinement, coziness, and intelligence”—were extracted. The results show that elderly users place great importance on affinity and warmth when using the center control panel, while also pursuing elegance in design and intelligence in functionality. Through morphological analysis, the study identified seven categories of design features and established relationships between emotional vocabulary and design features. Using entropy weighted-TOPSIS to rank and screen design features, the study ultimately identified the four most critical design features influencing elderly users’ emotional satisfaction: the center control screen, the co-driver entertainment screen, the control panel, and the center console outline. Based on this, the four core emotional vocabulary terms and key design features were input into a BPNN model to establish a mapping model between emotional vocabulary and key design features of the center console, and to predict the optimal aging-friendly center console design combination: center console Screen 2, co-driver entertainment screen 2, control panel 2, and center console outline 2.

### Analysis of elderly-oriented design for NEV center consoles based on KE

Based on the experimental results, the optimal combination for the aging-friendly design of the center console is the No. 2 center control screen, No. 2 co-driver entertainment screen, No. 2 control panel, and No. 2 center console outline, as shown in Fig. 8.

For elderly users, a simple design typically relies on basic geometric shapes with appropriately rounded corners, which provide a safe and reliable visual and tactile experience^60^. The No. 2 center control screen adopts a circular design with flowing lines and softened edges. This reduces the visual discomfort caused by sharp angles, particularly for elderly users. The circular structure naturally focuses the gaze, helping elderly users quickly lock onto the core information on the screen and reduce the visual search burden, which is particularly important for those with declining vision. The co-driver entertainment screen No. 2 primarily uses rounded rectangles, with screen size and proportions that are appropriate for elderly users’ visual range while ensuring clarity in information presentation. The control panel of the second model also uses a curved-rectangle design, providing ample operating space. This allows elderly users to quickly locate functions, thereby improving interaction efficiency. Finally, the overall outline of the second model’s center console is formed by continuous flowing curves, which avoid potential safety hazards from sharp edges. This larger curvature not only enhances visual aesthetics but also reduces the risk of collisions during operation.

From a holistic perspective, the most age-friendly central control panel design features fluid and uninterrupted lines. Visually, smooth curves reduce information complexity, enabling elderly users to process information more efficiently within their limited cognitive resources. Excessively complex or sharp edges can lead to information interference and visual strain^61^. Psychologically, sharp shapes often imply potential risks and create tension. In contrast, softened geometric forms are associated with safety, friendliness, and protection, thus evoking positive emotional experiences^62^. From a physiological perspective, as elderly users experience a decline in hand dexterity and fine motor skills, flat surfaces and smooth edges on buttons and controls not only facilitate grip and operation but also reduce the likelihood of accidental touches^63^. Overall, the soft and fluid design language effectively aligns with the perceptual and behavioral characteristics of elderly users across visual, psychological, and physiological dimensions, thereby enhancing the center console’s accessibility for the elderly.

When designing a central control panel for elderly users, it is essential not only to consider the form design characteristics derived from calculations but also to address four key emotional needs: approachability, elegance, warmth, and intelligence. Incorporating smooth, curved lines in the central control panel design, while avoiding sharp angles and rigid straight lines, can create a more friendly and inviting visual experience^64,65^; Designing a simple user interface with large text, clear icons, and high-contrast visual elements can help elderly users read information more easily^66^; In terms of color selection, it is advisable to use soft, warm, low-saturation neutral colors such as beige and light gray^67^. Compared to high-saturation or cold, hard colors, such color schemes can create a more stable and soothing usage atmosphere for elderly users^68,69^. In terms of material selection, older users generally prefer soft, warm-to-the-touch materials, which provide a sense of comfort and safety during use^70^. Using materials with a refined texture, such as genuine leather or suede, not only aligns with hand sensory characteristics but also creates a comfortable and reassuring atmosphere through their tactile qualities. Combining elegant lines with intricate decorative details can further enhance the sophistication and quality of the center console. Using warm-toned ambient lighting creates a cozy and comfortable driving environment^71^, providing comfortable illumination in low-light conditions while reducing glare and visual fatigue. In terms of interaction methods, the introduction of next-generation intelligent in-vehicle systems such as voice recognition and gesture control allows elderly users to perform functions such as navigation, music playback, or phone calls using natural language, thereby reducing reliance on complex touchscreen operations and alleviating cognitive and physical burdens. However, in actual usage scenarios, voice recognition accuracy may decrease due to environmental noise or accent differences. At the same time, touchscreen interfaces also present usability challenges for users with impaired vision. These issues may cause frustration among elderly users, thereby generating negative emotions toward the infotainment system experience. Future research could further validate the effectiveness and adaptability of multimodal interaction through contextualized experiments and provide empirical evidence for system optimization targeting elderly users.

Compared to previous studies that relied on subjective evaluations based on ergonomic principles or gray relational analysis (GRA), this study introduced entropy weighted-TOPSIS in the feature selection process, significantly reducing the interference of expert experience and subjective preferences. This allowed different design elements to be distinguished more clearly under objective weighting. For example, the comprehensive score for the center console outline (E5) (0.892) was significantly higher than that for the storage box (E7), and such a clear conclusion is often challenging to achieve in traditional expert-based evaluation systems. Additionally, the BPNN model demonstrated high stability and accuracy in predicting the mapping relationship between emotional words and design elements. Taking “sense of intelligence” as an example, the model achieved a fitting accuracy of R² = 0.994 and RMSE = 0.0219 in the training set, and maintained a high level (R² = 0.970) in the test set, indicating that the model could effectively capture the nonlinear relationship between elderly users’ emotions and design features.

### Application of entropy weighted-TOPSIS and BPNN in KE

In KE research, the core issue is how to objectively and scientifically determine the weighting relationship between emotional words and design features. In previous studies, Du et al.^72^used the fuzzy analytic hierarchy process (FAHP) to assess and rank the weights of characteristic elements of portable home air conditioners and validated them using the fuzzy comprehensive evaluation (FCE). Li et al^26^. employed the analytic hierarchy process (AHP) to rank the weights of emotional words. They then applied the Delphi method to deconstruct the spatial components of mobile exhibition spaces in community art galleries, thereby identifying the most critical components for the spatial structure. Shie et al^73^. utilized the fuzzy Kano model to filter emotional words, constructing a core vocabulary of emotional needs and mapping these core emotional needs to TRIZ engineering parameters. Gan et al.^74^used multiple linear regression analysis to determine the key morphological features of social robots that influence aesthetic appeal and emotional impression preferences. Llinares et al^75^. employed regression analysis and the Kano model to determine the weights of consumers’ emotional attributes in real estate purchase decisions. Zhou et al.^76^ applied Grey Relational Analysis to identify key visual elements that influence product form perception. However, these methods tend to rely on expert experience in feature evaluation, and the results are susceptible to subjective bias. Therefore, in order to determine the key design features of the center console, this study used the entropy weight-TOPSIS method to reduce the influence of subjective judgment and significantly improve the scientific validity of the results.

In the construction of emotional mapping models, traditional linear statistical methods, such as principal component analysis (PCA), quantitative theory type I (QT-I), and linear discriminant analysis (LDA), often fail to capture the complex nonlinear relationships between user emotions and design features. As artificial intelligence technology has advanced, methods such as Genetic Algorithms (GA)^77^, Support Vector Regression (SVR)^78,79^, Artificial Neural Networks (ANN)^31^, and Generative Adversarial Networks (GAN)^6^ have been applied to construct mapping relationships between product design features and user emotions^80^. Compared to these, GA involves high computational complexity and slow convergence rates. SVR is sensitive to parameter selection and performs poorly with large datasets. ANN, although powerful, lacks interpretability and is prone to overfitting. GAN may face instability and significant resource consumption during training.

Within the research framework of KE, this paper proposed a research method that combines entropy weighted-TOPSIS and BPNN. Compared with the above methods, the BPNN used in this study not only achieved nonlinear modeling but also showed strong predictive performance, with accuracy exceeding 0.92 across all four emotional dimensions. This was significantly better than the R² < 0.8 level commonly observed in linear methods. This result indicates that BPNN can accurately capture the changing trends of multi-dimensional emotional needs and demonstrates greater applicability in practical design predictions. It is noteworthy that the BPNN model in this study achieved relatively high performance metrics (training set R² = 0.994, test set R² = 0.970). Such high accuracy might raise concerns that the relationship between design features and user emotions could be predominantly linear or overly simple. However, users’ emotional responses to product morphology are generally considered to be non-linear and complex, often involving threshold effects that traditional linear models may struggle to capture. In this study, the enhanced performance is more likely attributable to two synergistic factors: first, the strong non-linear mapping capability of the BPNN enables it to decode the complex emotional structure of elderly users; second, the pre-screening of features using the entropy-weighted TOPSIS method reduces noise and redundant variables. These steps together provide a more focused and higher-quality input dataset, allowing the neural network to attain relatively high predictive precision while mitigating the risk of overfitting. Consequently, the feature selection of Entropy Weighted-TOPSIS and the emotional prediction of BPNN form a complementary relationship within the framework: the former simplifies the input feature set through objective evaluation, avoiding model redundancy, while the latter enhances prediction reliability through nonlinear learning. The combination of the two provides a new methodological reference for the application of affective engineering research in the field of aging-friendly design.

In terms of design significance, this method can not only be applied to the research of new energy vehicle central control panels but also provides a referenceable paradigm for aging-friendly products in other fields. By objectively extracting key elements and combining them with a nonlinear prediction model, an emotional mapping can be established. This allows designers to obtain reliable user emotional feedback at the early stages of product development, thereby reducing trial-and-error costs, shortening iteration cycles, and improving both design quality and user satisfaction in aging-friendly products.

## Conclusion

With the advancement of technology and the promotion of environmental protection policies, new energy vehicles have rapidly emerged in the market and have become a key factor in reducing carbon emissions and enhancing the sustainability of urban transportation. However, in the face of a rapidly growing elderly user population, traditional automotive designs often overlook the emotional needs of elderly users during the usage process. To achieve age-friendly design for the central control panel of new energy vehicles and meet the emotional needs of elderly users, this study proposes a research method that s Entropy Weight-TOPSIS and BPNN within the framework of affective engineering. This paper used FA to perform dimensionality reduction on the emotional intention vocabulary collected from elderly users regarding central control panel design, resulting in four core affective keywords. After deconstructing the design features of the central control panel, the Entropy Weight-TOPSIS method was used to screen key design features that significantly influence user emotions. BPNN was employed to establish a nonlinear relationship between user emotions and key design features, ultimately yielding the optimal combination of design elements for an age-friendly central control panel that aligned with the emotional preferences of elderly users. The main contributions of this study include:

(1) Previous studies have primarily focused on the exterior or interior design of automobiles, with limited systematic exploration of age-friendly design for the central control panel in new energy vehicles. This paper takes elderly users as the core target group, combining a large-scale emotional data analysis with sample surveys, thereby expanding the application of affective engineering in the field of age-friendly design.

(2) The Entropy Weight-TOPSIS method was used to objectively identify key design features, effectively avoiding the biases associated with traditional methods that rely on expert judgment.

(3) A Backpropagation Neural Network (BPNN) was employed to replace traditional linear analysis methods, establishing a nonlinear mapping relationship between elderly users’ emotions and design features. This approach yielded the optimal combination of age-friendly design elements for new energy vehicle center consoles that align with the emotional preferences of elderly users.

Nevertheless, this paper still has some limitations that need to be improved:

(1) This paper focused primarily on the morphological characteristics of the central control panel in new energy vehicles. However, the emotional experience of elderly users when using new energy vehicles is not only influenced by morphological factors but also by various other factors such as interface design, seat comfort, and multimodal interaction. Future research could further expand the scope of study by combining physical morphology with interface design, comprehensively considering the aforementioned influencing factors, and establishing a unified evaluation system to deeply explore the mechanisms underlying the impact of new energy vehicle central control panel design on elderly users’ experience and emotional responses.

(2) This study employed methods such as entropy-weighted TOPSIS and BPNN in emotional needs identification to minimize subjective bias in the analysis process. However, the analysis failed to adequately account for differences among elderly groups in terms of health status, technical proficiency, gender differences, and lifestyle habits. Future research could incorporate analyses of different elderly groups into the user sample to construct more targeted emotional needs profiles. Additionally, by combining physiological measurement techniques such as electroencephalography (EEG) and eye tracking with multi-modal user experience testing methods like user interviews and behavioral path analysis, the accuracy and objectivity of emotional needs recognition could be enhanced.

(3) The participant sample in this study consisted of elderly users (*N* = 153). While this sample size was statistically acceptable for the EFA used for data preprocessing (KMO = 0.678), it falls below the generally recommended threshold for conducting a stable Confirmatory Factor Analysis (CFA) (e.g., *N* > 200). Consequently, this study focused primarily on exploring affective structures to optimize the predictive model inputs. Future research should aim to further verify the stability and theoretical validity of these Kansei structures by employing CFA based on a larger participant sample.

(4) This study used the central control panel of new energy vehicles as a case study and employed BPNN to construct an emotion mapping model. Although the model achieved high predictive accuracy in the current experiment, it was trained on a relatively small sample dataset (60 samples). Therefore, the high performance metrics should be interpreted with caution regarding potential overfitting risks, and the model’s generalization ability may be limited when applied to broader datasets. In the future, this design model could be extended to various fields such as smart home devices, wearable devices, and medical assistive devices. Simultaneously, the scale of the training sample could be expanded, and more diverse feature data could be introduced to explore additional data-driven methods, such as deep learning, other machine learning algorithms, and hybrid models. By combining cross-validation or an independent test set, the stability of predictions regarding user emotions and behavioral responses could be further enhanced.

In summary, this study has developed a set of methods for aging-friendly design of the central control panel in new energy vehicles, emphasizing the importance of incorporating emotional design elements into the design of the central control panel. The research findings provided a theoretical foundation and practical guidance for aging-friendly optimization of in-vehicle human-machine interaction, holding positive implications for promoting human-centered intelligent cockpit design. As technology and design continue to evolve, the methods proposed in this study are expected to be further optimized and refined, offering a more robust foundation for product innovation and optimization.

## Supplementary Information

Below is the link to the electronic supplementary material.

Supplementary Material 1

## Data Availability

The datasets used and/or analyzed during the current study available from the corresponding author on reasonable request.
