# Supplementary material for: Emotional design of new energy vehicle center consoles for elderly users based on entropy weighted TOPSIS and BPNN
Source: Sci Rep. 2025 Dec 8;16:1658. doi: 10.1038/s41598-025-31226-4 (PMC12800025; doi:10.1038/s41598-025-31226-4)
Supplement: Supplementary file 1 — Supplementary Material 1 [file 41598_2025_31226_MOESM1_ESM.pdf]

Supplementary Table S1. Emotional evaluation matrix for the center console of NEV

NO.	Comfortable	Simple	Practical	Friendly	Cozy	Technological	Elegant	Bright	Innovative	Advanced
1	4.62	4.37	4.47	4.65	4.61	4.84	4.37	4.61	4.60	4.41
2	4.60	4.43	4.57	4.63	4.62	5.01	4.42	4.57	4.56	4.61
3	4.70	4.41	4.53	4.58	4.76	4.92	4.44	4.83	4.73	4.43
4	4.68	4.61	4.35	4.46	4.36	4.78	4.46	4.61	4.46	4.35
5	4.66	4.47	4.48	4.32	4.81	4.68	4.46	4.63	4.70	4.59
6	4.72	4.52	4.49	4.69	4.76	4.79	4.46	4.67	4.75	4.56
7	4.62	4.59	4.57	4.56	4.81	4.86	4.46	4.76	4.46	4.67
8	4.64	4.34	4.54	4.61	4.83	4.83	4.47	4.69	4.51	4.78
9	4.65	4.47	4.52	4.57	4.52	4.88	4.48	4.86	4.57	4.43
10	4.69	4.42	4.50	4.68	4.52	4.73	4.49	4.26	4.43	4.55
11	4.79	4.54	4.52	4.61	4.64	4.59	4.49	4.76	4.50	4.48
12	4.69	4.52	4.37	4.56	4.67	4.97	4.50	4.73	4.77	4.48
13	4.63	4.54	4.55	4.71	4.54	4.94	4.51	4.46	4.69	4.71
14	4.75	4.62	4.54	4.73	4.53	4.86	4.51	4.29	4.83	4.58
15	4.65	4.37	4.35	4.55	4.56	4.74	4.52	4.75	4.43	4.57
16	4.56	4.59	4.38	4.48	4.64	4.89	4.54	4.78	4.72	4.57
17	4.60	4.62	4.50	4.52	4.75	4.89	4.54	4.71	4.84	4.68
18	4.71	4.80	4.64	4.78	4.54	4.57	4.56	4.65	4.35	4.50
19	4.68	4.62	4.39	4.55	4.64	4.75	4.59	4.74	4.50	4.56
20	4.71	4.54	4.39	4.69	4.77	4.97	4.59	4.62	4.66	4.50
21	4.45	4.58	4.32	4.46	4.86	4.74	4.61	4.97	4.61	4.62
22	4.83	4.72	4.59	4.65	4.57	4.92	4.61	4.64	4.74	4.63
23	4.68	4.31	4.29	4.39	4.85	4.76	4.62	4.75	4.22	4.68
24	4.65	4.78	4.59	4.71	4.59	4.89	4.62	4.70	4.69	4.77
25	4.65	4.39	4.47	4.50	4.81	4.73	4.63	4.67	4.61	4.55
26	4.52	4.44	4.44	4.38	4.66	4.69	4.64	4.61	4.78	4.66
27	4.83	4.49	4.52	4.66	4.75	4.80	4.64	4.71	4.36	4.50
28	4.58	4.49	4.48	4.66	4.76	4.80	4.65	4.66	4.45	4.64
29	4.77	4.30	4.58	4.65	4.58	4.77	4.65	4.77	4.65	4.58
30	4.58	4.28	4.25	4.32	4.65	4.80	4.66	4.52	4.64	4.54
31	4.70	4.51	4.45	4.48	4.80	4.74	4.66	4.80	4.52	4.67
32	4.67	4.43	4.47	4.55	4.66	4.92	4.66	4.55	4.61	4.85
33	4.49	4.45	4.38	4.59	4.76	4.82	4.67	4.81	4.68	4.50
34	4.62	4.46	4.52	4.69	4.91	4.64	4.67	4.83	4.61	4.57
35	4.59	4.43	4.55	4.55	4.50	4.91	4.67	4.31	4.47	4.76
36	4.45	4.30	4.32	4.29	4.76	4.83	4.68	4.79	4.54	4.46
37	4.68	4.59	4.41	4.51	4.61	4.83	4.69	4.52	4.59	4.64

38	4.66	4.41	4.52	4.72	4.59	4.72	4.69	4.71	4.58	4.72
39	4.66	4.79	4.61	4.82	4.68	4.74	4.69	4.58	4.67	4.76
40	4.50	4.40	4.50	4.73	4.70	4.83	4.70	4.73	4.82	4.83
41	4.78	4.81	4.59	4.78	4.70	4.89	4.70	4.64	4.59	4.65
42	4.75	4.50	4.47	4.61	4.73	4.88	4.71	4.67	4.62	4.62
43	4.66	4.30	4.47	4.61	4.75	4.71	4.71	4.62	4.68	4.74
44	4.64	4.41	4.51	4.61	4.90	4.85	4.71	4.91	4.36	4.57
45	4.87	4.98	4.71	4.80	4.64	4.80	4.72	4.61	4.62	4.86
46	4.55	4.89	4.67	4.71	4.62	4.81	4.73	4.60	4.47	4.68
47	4.57	4.29	4.31	4.45	4.73	4.71	4.74	4.85	4.47	4.65
48	4.65	4.29	4.39	4.31	4.82	4.82	4.75	4.78	4.61	4.69
49	4.65	4.59	4.42	4.65	4.66	4.87	4.75	4.51	4.61	4.81
50	4.54	4.43	4.49	4.65	4.77	4.91	4.75	4.87	4.82	4.59
51	4.57	4.50	4.47	4.63	4.86	4.85	4.76	4.75	4.83	4.74
52	4.68	4.41	4.50	4.64	4.54	4.90	4.76	4.68	4.82	4.59
53	4.64	4.54	4.58	4.76	4.58	4.64	4.76	4.64	4.76	4.85
54	4.59	4.45	4.55	4.71	4.52	4.80	4.77	4.77	4.57	4.74
55	4.59	4.57	4.46	4.66	4.44	4.66	4.80	4.26	4.61	4.71
56	4.71	4.59	4.59	4.75	4.69	4.83	4.80	4.64	4.60	4.66
57	4.79	4.82	4.66	4.86	4.43	4.87	4.80	4.28	4.80	4.76
58	4.61	4.48	4.46	4.75	4.71	4.55	4.83	4.72	4.48	4.78
59	4.85	4.91	4.68	4.75	4.75	4.95	4.90	4.64	4.65	4.85
60	4.74	4.50	4.55	4.70	4.92	4.85	4.91	4.52	4.82	4.74

Supplementary Table S2. Elderly user emotional dataset

NO.	Affinity	Refinement	Coziness	Intelligence
1	4.33	4.72	4.54	3.80
2	3.99	3.91	4.47	4.49
3	4.71	3.82	4.37	3.87
4	4.22	4.42	3.39	3.89
5	4.69	3.74	4.35	3.73
6	4.29	4.60	4.47	3.84
7	4.04	4.33	3.67	4.29
8	4.65	3.84	4.31	3.78
9	3.95	3.88	4.37	4.39
10	4.24	4.55	4.39	3.76
11	4.83	3.49	4.48	4.10
12	4.37	4.65	4.55	3.88
13	4.27	4.25	3.61	3.85
14	3.46	4.21	4.28	4.41

15	4.91	3.54	4.21	4.03
16	4.64	4.55	4.24	4.05
17	4.05	4.30	3.67	4.17
18	4.63	4.18	4.45	3.80
19	3.44	4.11	4.34	4.33
20	4.79	3.50	4.55	4.19
21	3.89	4.08	4.31	4.69
22	4.35	4.18	4.41	4.13
23	4.98	3.45	4.41	3.88
24	4.42	4.25	4.42	4.16
25	3.87	4.66	3.70	3.84
26	4.52	4.33	4.39	4.13
27	3.98	3.82	4.42	4.47
28	4.41	4.27	4.44	4.19
29	4.38	4.45	3.35	4.05
30	4.73	4.26	4.43	3.84
31	3.77	3.88	4.38	4.85
32	4.77	3.38	4.27	4.21
33	4.55	4.39	4.41	4.17
34	3.83	3.73	4.29	4.36
35	3.89	4.68	3.63	3.79
36	4.44	4.30	4.46	4.16
37	4.23	4.21	3.63	3.80
38	3.88	4.71	3.68	3.88
39	4.84	3.52	4.56	4.20
40	4.45	4.22	4.47	4.24
41	4.02	4.31	3.61	4.13
42	4.65	4.20	4.54	3.91
43	3.54	4.24	4.45	4.48
44	4.46	4.32	4.60	4.24
45	4.32	4.57	4.38	3.87
46	4.23	4.33	3.65	3.79
47	4.10	4.41	3.69	4.24
48	4.62	4.47	4.23	3.97
49	4.87	3.54	4.43	4.14
50	4.62	4.17	4.45	3.86
51	3.57	4.17	4.38	4.47
52	4.04	4.38	3.67	4.19
53	4.21	4.63	4.46	3.80
54	3.74	3.86	4.48	4.87
55	5.10	3.42	4.35	3.74

56	4.47	4.19	4.47	4.22
57	3.92	3.88	4.47	4.50
58	3.94	4.68	3.69	3.82
59	3.74	3.83	4.35	4.81
60	5.00	3.66	4.19	4.06

Supplementary Table S3. Kansi semantic evaluation matrix

NO.	E1	E2	E4	E5	Affinity	Refinement	Coziness	Intelligence
1	1	1	1	1	4.33	4.72	4.54	3.80
2	1	1	2	1	3.99	3.91	4.47	4.49
3	2	7	3	2	4.71	3.82	4.37	3.87
4	5	7	12	6	4.22	4.42	3.39	3.89
5	5	7	12	3	4.69	3.74	4.35	3.73
6	5	7	12	6	4.29	4.60	4.47	3.84
7	5	7	4	6	4.04	4.33	3.67	4.29
8	5	7	1	3	4.65	3.84	4.31	3.78
9	3	7	1	3	3.95	3.88	4.37	4.39
10	5	7	1	3	4.24	4.55	4.39	3.76
11	5	7	12	9	4.83	3.49	4.48	4.10
12	5	7	5	2	4.37	4.65	4.55	3.88
13	5	7	3	7	4.27	4.25	3.61	3.85
14	5	7	4	7	3.46	4.21	4.28	4.41
15	5	7	12	4	4.91	3.54	4.21	4.03
16	3	7	12	9	4.64	4.55	4.24	4.05
17	3	7	12	7	4.05	4.30	3.67	4.17
18	5	7	12	6	4.63	4.18	4.45	3.80
19	5	2	12	5	3.44	4.11	4.34	4.33
20	3	7	6	2	4.79	3.50	4.55	4.19
21	5	3	12	7	3.89	4.08	4.31	4.69
22	1	7	7	9	4.35	4.18	4.41	4.13

23	6	7	7	13	4.98	3.45	4.41	3.88
24	5	7	12	6	4.42	4.25	4.42	4.16
25	12	7	3	5	3.87	4.66	3.70	3.84
26	8	7	12	4	4.52	4.33	4.39	4.13
27	5	3	12	10	3.98	3.82	4.42	4.47
28	3	7	12	8	4.41	4.27	4.44	4.19
29	9	7	12	7	4.38	4.45	3.35	4.05
30	7	7	8	12	4.73	4.26	4.43	3.84
31	5	1	12	11	3.77	3.88	4.38	4.85
32	10	5	10	12	4.77	3.38	4.27	4.21
33	11	4	9	13	4.55	4.39	4.41	4.17
34	5	7	12	12	3.83	3.73	4.29	4.36
35	12	7	11	5	3.89	4.68	3.63	3.79
36	5	7	12	6	4.44	4.30	4.46	4.16
37	5	7	4	9	4.23	4.21	3.63	3.80
38	12	7	3	8	3.88	4.71	3.68	3.88
39	1	7	12	2	4.84	3.52	4.56	4.20
40	11	7	10	1	4.45	4.22	4.47	4.24
41	5	7	12	9	4.02	4.31	3.61	4.13
42	5	7	12	12	4.65	4.20	4.54	3.91
43	9	7	12	10	3.54	4.24	4.45	4.48
44	9	1	12	11	4.46	4.32	4.60	4.24
45	5	3	12	12	4.32	4.57	4.38	3.87
46	13	7	1	13	4.23	4.33	3.65	3.79
47	5	7	1	10	4.10	4.41	3.69	4.24
48	8	7	10	6	4.62	4.47	4.23	3.97
49	14	7	12	10	4.87	3.54	4.43	4.14
50	3	7	11	12	4.62	4.17	4.45	3.86
51	9	7	1	11	3.57	4.17	4.38	4.47

52	7	7	12	9	4.04	4.38	3.67	4.19
53	12	7	5	6	4.21	4.63	4.46	3.80
54	13	6	3	6	3.74	3.86	4.48	4.87
55	5	7	3	11	5.10	3.42	4.35	3.74
56	5	7	5	10	4.47	4.19	4.47	4.22
57	4	7	3	9	3.92	3.88	4.47	4.50
58	9	7	12	12	3.94	4.68	3.69	3.82
59	7	7	1	3	3.74	3.83	4.35	4.81
60	5	7	12	6	5.00	3.66	4.19	4.06
